# Students’ Learning Behaviour in Programming Education Analysis: Insights from Entropy and Community Detection

**DOI:** 10.3390/e25081225

**Published:** 2023-08-17

**Authors:** Tai Tan Mai, Martin Crane, Marija Bezbradica

**Affiliations:** 1 School of Computing, Dublin City University, Collins Ave Ext, Whitehall, D09 Y074 Dublin, Ireland; martin.crane@dcu.ie (M.C.); marija.bezbradica@dcu.ie (M.B.); 2 ADAPT Center for Digital Content Technology, D02 PN40 Dublin, Ireland

**Keywords:** entropy, learning behaviours, learning analytics, educational data mining, community detection, random matrix theory

## Abstract

The high dropout rates in programming courses emphasise the need for monitoring and understanding student engagement, enabling early interventions. This activity can be supported by insights into students’ learning behaviours and their relationship with academic performance, derived from student learning log data in learning management systems. However, the high dimensionality of such data, along with their numerous features, pose challenges to their analysis and interpretability. In this study, we introduce entropy-based metrics as a novel manner to represent students’ learning behaviours. Employing these metrics, in conjunction with a proven community detection method, we undertake an analysis of learning behaviours across higher- and lower-performing student communities. Furthermore, we examine the impact of the COVID-19 pandemic on these behaviours. The study is grounded in the analysis of empirical data from 391 Software Engineering students over three academic years. Our findings reveal that students in higher-performing communities typically tend to have lower volatility in entropy values and reach stable learning states earlier than their lower-performing counterparts. Importantly, this study provides evidence of the use of entropy as a simple yet insightful metric for educators to monitor study progress, enhance understanding of student engagement, and enable timely interventions.

## 1. Introduction

In recent years, computer programming and related domains have garnered significant attention, primarily due to the escalating demand for information and communication technology (ICT) skills in various job markets. As a result, the incorporation of programming courses has become a component of most ICT-related degree programs. However, this popularity can be a double-edged sword. The more students are drawn to programming, the greater the likelihood of a spread of abilities in a class. Therefore, to ensure the best practices in pedagogy within the ICT field, it is crucial to focus on enhancing students’ engagement and optimising their individual learning processes regarding the learning materials provided [1].

Despite the aforementioned increasing demand for ICT professionals and the emphasis on programming education, numerous studies have highlighted a concerning issue. There is evidence that many students find computer programming challenging [2], potentially resulting in high dropout rates in introductory programming courses [3]. These rates have been reported to vary significantly, ranging from 0% to a staggering 91%, with an average of 28% reported in introductory programming modules across 161 universities worldwide [3]. Such statistics underscore the importance of investigating and addressing the factors contributing to the high dropout rates in introductory programming courses. By understanding the challenges faced by students and implementing effective strategies to remediate them, educators and institutions can improve the learning experience and enhance student retention in these critical courses.

In the main, efforts to reduce dropout rates in programming education involve identifying the specific struggling points that students encounter during the learning process. Factors such as the steep learning curve [4], lack of prior programming experience [5], inadequate support systems [4], and ineffective teaching methodologies [6] have been identified as potential contributors to student disengagement and subsequent dropout. Therefore, educational institutions need to develop comprehensive approaches that tackle these challenges.

One potential approach [7] is to provide students with timely and constructive feedback on their assignments and projects, which can significantly impact their motivation and progress. Regular assessments and feedback sessions serve to allow students to track their development, identify areas for improvement, and receive guidance from instructors. Such an iterative feedback loop nurtures a sense of accomplishment and encourages students to persevere, ultimately reducing the likelihood of dropout. However, where student populations are high, a spread of abilities is present, or class sizes are correspondingly large, conventional methods of monitoring individual student behaviours and providing personalised feedback can become considerably challenging. Traditional pedagogical approaches, such as individual counselling or personalised written feedback, may not be scalable or efficient in such circumstances [8]. This situation emphasises the necessity for innovative solutions that can effectively address this dichotomy between increasing class sizes and the persistent need for individualised student tracking and feedback.

The evolution of educational technologies has facilitated the acquisition of a wealth of learning data generated by course participants within computer-supported environments [9]. These advanced systems possess the capability to autonomously document substantial quantities of interaction data at granular levels, encompassing, for instance, mouse and keyboard event-specific activities. The ensuing log data hold significant potential for educators; they can be harnessed to elucidate valuable insights into the ongoing learning trajectories of individual students [10]. Understandings of such nuances can, in turn, significantly contribute to the enhancement of both teaching methodologies and learning outcomes [11], thereby adding a new dimension to educational research and pedagogical practice.

Learning behaviour data can be leveraged in multiple ways for educational research, with an aim to dynamically monitor students’ learning progress during the studying time. One approach employs machine learning techniques, such as clustering and classification, to predict student outcomes or to identify patterns of student behaviours [12]. Another approach utilises sequential pattern mining to discover common sequences of learning behaviours and to predict future actions [13]. Additionally, social network analysis can be used to understand the interactive behaviours within learning communities [14]. However, the utility of these techniques might be challenged by the curse of dimensionality; the problem arises when working with high-dimensional data [15]. As educational data often include a wide array of variables ranging from demographic information to granular clickstream data, the high-dimensional space may cause reduced interpretability of analyses.

In an attempt to incorporate this human interaction, there has been emerging attention to adopting concepts from disparate disciplines to elucidate the link between human behaviours and learning patterns. One such concept is *entropy*, a construct originally rooted in thermodynamics but which has gained attention in behavioural studies [16]. Entropy, in essence, quantifies the randomness or uncertainty inherent in a system [17]. In an educational context, this system may be viewed as the various learning activities that students engage in throughout their academic journey.

These learning activities can be collected and represented as a set of distinct engagements, each with varying degrees of intensity and frequency. Based on the concept of entropy, we propose a novel approach to synthesise these multidimensional data into a single comprehensive metric. This entropy-based metric amalgamates the diverse range of students’ learning activities, thereby offering a holistic view of their academic engagement.

In essence, our key objective in utilising the concept of entropy as a metric is to simplify the process of assessing individual and collective student engagement for educators. By offering a single measure that encapsulates a variety of learning behaviours across student communities, we aim to expedite the process of tracking and understanding student engagement. Consequently, this entropy metric could provide educators with timely insights, enabling them to tailor their teaching strategies and interventions based on the dynamic learning landscape captured and target interactions towards those student groups whose need is greater. This paper is inspired by and marks the development of [18], where the authors have proven the potential of using learning behavioural data in monitoring student studying progress.

In addition, specifically, the research objective is to answer the following research questions:

- RQ1: Can the concept of entropy be used to represent the individual learning behaviour of students?
- RQ2: From an entropy point of view, can we see a difference in the learning behaviour between the higher- and lower-performing communities of students?
- RQ3: Can entropy-based metrics be used as a dynamic index to monitor students’ learning progress during the studying time?

To conduct the research in this paper, we utilise the datasets that contain 391 university Software Engineering students participating in a programming course during the three academic years 2018, 2019, and 2020.

The rest of the paper is organised as follows: Section 2 discusses the related work. Section 3 briefly describes the context of the study and the datasets. Section 4 refers to research methods. Section 5 and Section 6 provide details of the results and discussion, followed by the conclusion in Section 7.

## 2. Related Works

### 2.1. Analytics of Learning Behaviours

Learning analytics (LA) and educational data mining (EDM) represent two distinct yet interrelated domains that leverage the principles of data mining specifically tailored for educational contexts [19]. They offer a proven methodological approach to discern patterns of usage behaviours and engagements, which are typically derived from user interactions within a learning system. Their application consequently equips educators with thorough insights into the mechanisms of student learning [20]. For example, EDM/LA may be utilised to identify less common student behaviours by examining the correlations between online activities and final grade outcomes. This allows for an in-depth understanding of how specific behavioural patterns may influence academic performance [21].

The connection between learning behaviours and student performance has been a subject of investigation in numerous studies [22]. One illustrative example of this is a pilot study conducted with a small cohort of learners [23]. In this study, the authors orchestrated a web programming course involving 13 participants, facilitated through what the authors term a “web-based programming assisted system for cooperation”. Despite the limited scope of this preliminary study, which drew data solely from an experimental class, it presented preliminary evidence of a correlation between learning behaviour styles (such as complete independence, imitation, and self-improvement through assistance) and learning outcomes in programming education. This initial finding underscores the potential value of further exploration in this area.

In a separate study [24], researchers have formulated metrics intended as formative assessment tools, designed to dissect students’ learning patterns. These metrics have been predominantly applied to practical activities like coding and resolving programming tasks. However, the scope of this research could be broadened by incorporating a wider range of learning activities integral to the process of learning programming, such as the study of lecture notes and lab sheets, in addition to coding exercises.

On the other hand, identifying patterns of student data that show similarities in their features based on clustering techniques has been proven to establish useful inferences [25]. For example, in [26,27], the authors could detect groups of students who have shown similar learning characteristics based on visiting content on the webpages of the LMS. This finding can be used to recommend preferred learning activities and resources to students. Furthermore, behaviour-related features, such as total time spent on theoretical and practical contents and forums, can be used to find clusters of procrastination and thus to focus students, e.g., by setting intermediate time goals [28]. The authors in [29,30] created a network structure of undergraduate courses and applied community detection algorithms to identify the contributions of the courses to students’ learning pathways. Such findings can support the understanding of students’ behaviours in various learning situations [31] and the identification of potential dropouts at the early stage of the academic year [28].

Community detection (CD) refers to the procedure of detecting groups of interacting nodes in a graph based on their structural properties [32]. In other words, community detection can be considered as a clustering technique that can be applied to the graph to detect communities with similar properties and behaviours so that they can be grouped together. Many algorithms for community detection have been developed [33] and applied to a variety of disciplines, such as social networks [34] and economics [35]. With respect to the educational domain, the application of CD has been limited and is usually in the form of social network analysis. For example, a graph can be constructed based on the data about communications between students, e.g., asking questions and giving answers under each topic of the study [36], or discussions via learning forums [37] in online learning platforms, where students within the same community show a higher level of communication with each other than with students outside the community.

In order to investigate the learning resource usage, in this paper, we follow the community detection approach, using extracted behavioural features from the logs. In particular, we construct a network structure based on students’ learning behavioural data to produce more logical and coherent communities in terms of their learning performance. The network structure of undergraduate courses and their contributions to students’ learning pathways have been investigated using the community detection approach and minimum spanning tree [29,30], which are similar to the approach of this research. However, the authors of both studies merely considered the courses’ grades from a relatively small number of students. We would argue that more aspects of student learning, e.g., student learning behaviours, can be included to deliver more insightful results.

### 2.2. The Concept of Entropy in Human Behaviour Studies

In the area of human behaviour studies, the concept of entropy has been shown to provide valuable insights into the complexity, variability, and unpredictability of human actions and decision making processes [16]. Entropy, originating from information theory, quantifies the level of disorder or uncertainty within a system [17].

Applied to human behaviour, entropy can be used to measure the degree of randomness or diversity in individuals’ choices, preferences, and actions [38]. By analysing entropy in human behaviour, researchers can gain a deeper understanding of the underlying patterns, motivations, and dynamics that influence human decision making. For example, a study in [39] found evidence for entropy maximisation in human free choice behaviour. The authors found that individuals have a preference for choosing options that provide further choices, even when the additional choice provides no gain in reward or even a sacrifice of reward [39]. This behaviour has been difficult to account for with classical economic decision models [40] that consider decisions on the basis of expected utility alone. Inferential accounts of decision making have considered the value of occupying states with more options available as a means of entropy maximisation, in addition to utility maximisation [39]. In [41], the authors applied a method using the concept of transfer entropy [42] to estimate synchronised behaviour and interpersonal relationships in human communication. The study found that transfer entropy could be used to identify the causal relationship between two people (leader and follower) during a cooperative task.

Specifically in the fields of education and learning analytics, the concept of entropy can be used to measure and understand students’ progress by quantitatively measuring the difference between the content to be learned, the tutors’ expectation of understanding, and the students’ knowledge [43]. In another study [44], an entropy-based classification method has been proposed to optimise the level of personalisation in learning management systems. Entropy is also used in education systems to transform an individual’s learning experience by providing a personalised learning environment that is tailored to their needs [45]. However, the utilisation of entropy in the domains of educational data mining and learning analytics appears to be constrained and has not yet reached its full potential.

Regarding students’ learning behaviours and their impact on academic performance, previous research [18] introduced a clustering method based on community detection, successfully establishing relationships between groups of students exhibiting similar learning behaviours and their corresponding academic achievements. However, the data aggregation in this method was limited to a weekly basis, potentially leading to the loss of valuable information concerning students’ studying schedules, particularly their behaviours on studying and non-studying days within the week. Additionally, the interpretation of these student behaviours within the identified groups appears to be intricate due to the high dimensionality in data features of the learning behaviour datasets, which was not the focus of the study [18]. To address these challenges, this paper presents an advancement of the previous study. Particularly, we propose a novel approach, based on Shannon entropy, with entropy-based metrics extracted from learning behavioural data features. The metrics are used to analyse and compare students’ learning behaviours from different angles.

The objectives of this paper are twofold: (1) to confirm the relationship between students’ learning behaviours and learning performance from an entropy perspective, and (2) in combination with a community detection approach, to employ these entropy-based metrics as simple yet comprehensive and effective indicators for highlighting the main characteristics of students in each detected community of students, thereby circumventing the need for the explanation of diverse data features.

With respect to datasets, our research has been carried out on a large volume of learning log data automatically collected during the study from our bespoke online learning platform from real university programming classes over three academic years. This aims to avoid any sources of experimental setup bias. The learning behavioural data are also analysed at the lower granularity level, i.e., observing the students’ learning behaviour using entropy-based metrics on a daily basis instead of a weekly basis, as can be seen in [18]. By incorporating entropy-based metrics at a fine-grained level of data, we expect to provide researchers and educators with a more insightful approach to examining learning behaviours and their implications on academic performance, potentially contributing to more targeted and effective educational interventions.

### 2.3. The Effect of COVID-19 on Learning Behaviours

The COVID-19 pandemic has been identified as a significant factor influencing higher-education students, affecting their learning behaviours and overall satisfaction [46]. The crisis has caused a shift in pedagogical methods and learning paradigms [47]. With the transition to home-based or hybrid learning, students may find themselves with an increased temporal capacity to engage in diverse activities, such as physical exercises, exploring supplementary educational resources, or even writing poetry. This novel learning context, however, necessitates a set of self-regulation skills to manage their learning trajectory effectively [48]. Without these skills, learners may encounter negative outcomes.

In certain educational environments, such as a prosthodontic programme, students have manifested the generally negative effect of online learning [49], which can trigger psychological distress [50]. An interesting shift has been noted in the context of autonomous learning during the COVID-19 lockdown, where students have been observed to adopt more consistent learning strategies as opposed to restricting their studies to particular weekdays [51]. Therefore, this research also utilises a student dataset collected during the lockdown to validate the potential of using entropy-based metrics to observe these pandemic-induced alterations in learning behaviour.

## 3. Data Collection

### 3.1. Context of the Study

This study utilises three datasets encompassing the learning behaviour of students and their performance in an introductory programming course, referred to as *Module*, offered within the Software Engineering program at a medium-sized metropolitan university. The datasets comprise Module-2018 and Module-2019, conducted prior to the onset of the COVID-19 pandemic, and Module-2020, conducted during the pandemic lockdown. Pre-COVID-19 courses were delivered through a blend of conventional and online instructional methods, with students attending lecture sessions physically in lecture halls and engaging in learning activities via a customised online platform. Conversely, during the pandemic lockdown, students participated in remote learning from their respective homes, and it is assumed that all students possessed equal access to the online learning system.

Throughout the study duration, students were provided with weekly learning material items as part of the course curriculum. These course items encompassed general course information, lecture notes, labsheets, and programming tasks. During lecture sessions, students were expected to peruse the lecture notes, while, in lab sessions, they were required to follow instructions and examples provided in the labsheets and complete designated programming tasks. Subsequently, students submitted their solutions to the tasks, which were subject to automated testing by the system. The course materials were presented in the form of web pages accessible via the bespoke online learning system.

A pivotal component of the assessment was the final lab exam, which students were expected to approach diligently by competently addressing all assigned programming tasks. Based on their performance in this exam, students were categorised into two groups: those achieving a grade of less than 40 out of 100 were identified as “lower-performing”, while students scoring 40 or above were designated as “higher-performing”.

In this context, we establish a formal representation for the course material items, categorised by their material type (i.e., General, Lecture, Labsheet, and Practice) and linked to the corresponding week. For instance, Labsheet1 signifies the labsheet associated with week 1. As for the general documents, encompassing course information and technical instruction notes, they are denoted as General. Notably, the student interactions with these items, such as mouse clicking or scrolling on a lecture note, are automatically logged and stored in the database. Concise details pertaining to the collected data are presented in Table 1 for reference.

All data processing procedures have been meticulously carried out in strict adherence to the General Data Protection Regulation (GDPR) and ethical guidelines. To ensure confidentiality and privacy, personal identity data have been subjected to anonymisation. Furthermore, the comprehensive collection and utilisation of the data have received official approval from the Research Ethics Committee of the respective universities where the data were acquired.

### 3.2. Transition Frequency Features

In this paper, we utilise the concept of *transition frequency features*, i.e., the number of occurrences that a student moves from one event on a course item to another event. The concept of *transition* refers to the phenomenon that a student switches from an action on a learning material item to the next action on the same or another learning material item when interacting with the learning system. For example, when the student s1 scrolls down the page of the lecture notes in *Lecture1*, then clicks on the link to open the page *Labsheet1*, the following transitions can be recorded, i.e., *Lecture1-Lecture1* and *Lecture1-Labsheet1*. Please note that the two events can be on the same item or two different items. We use the term *transition* to denote this phenomenon of moving between consecutive events.

The *transition frequency features* can be arranged as *transition–student data matrix* where the rows refer to *transition frequency features* and the columns are the data for the students. An example of a transition data matrix of an event log can be seen in Table 2. The value of *Lecture1-Labsheet1* for student s2 equals to 14 indicates that student s2 performed an event 14 times on *Lecture1* directly before the next event on *Labsheet1*. Please note that, if the two materials are the same, e.g., *Lecture1-Lecture1*, the transition reflects a loop in the learning process, i.e., the student keeps working on the same course item *Lecture1*.

The *transition frequency features* from Module over the three academic years (i.e., 2018, 2019, and 2020) have been extracted. Three datasets are extracted from the event logs, namely Module-2018, Module-2019, and Module-2020. A summary of the extracted datasets can be seen in Table 3.

## 4. Research Methodology

In this research, we employed an innovative approach to extracting entropy-based metrics from students’ learning log data, utilising the datasets and transition frequency features that had been collected. Then, these extracted metrics were used in analysing differences in students’ learning behaviours across different student communities and academic years.

However, we have observed that a naïve approach based solely on students’ exam marks could potentially lead to misleading results. There were instances where students with lower academic results exhibited learning patterns akin to those of successful students and vice versa. Additionally, the noise inherent in such activity log data and the trend effect—where all students in a class may follow the same study pathway—could confound the interpretation of our findings.

To mitigate these issues, we have adopted a method reported in the literature [18] that combines the principles of random matrix theory and community detection. This methodology effectively filters the noise and trend effects in the log data, enabling a more reliable categorisation of students into distinct communities.

Having determined these student communities via this network-based clustering method, we have proceeded to compare the entropy-based metrics across these groups. Given that we cannot assume a normal distribution for our data, we adopted non-parametric statistical tests, specifically the Mann–Whitney U test, for this comparative analysis.

The technical details of our methodology, including the data collection, extraction of entropy-based metrics, community determination, and statistical analysis, are delineated in the subsequent subsections of this paper. The overarching objective of our methodological approach was to ensure robustness in our findings and provide meaningful insights into students’ learning behaviours, taking into account the unique circumstances of each academic year.

### 4.1. Entropy of Learning Behaviour

In our approach, we propose a method for computing the learning behavioural entropy grounded in the principles of Shannon entropy [52]. Specifically, consider *L* as the set of learning items utilised by a student during the course. Let $p(x_{ij})$ be the probability that a student transitions from learning item *i* to learning item *j*. For example, in Table 2, assuming that there are only three types of transition in the first column for each student, with the learning item *i* as Labsheet1, learning item *j* as Practice1 (row 3 in Table 2), one can observe $x_{ij}=12$ and compute the probability $p(x_{ij})=12/16=0.75$.

For a student *k*, the entropy of that student’s learning behaviour—denoted $E_{k}$—can be computed as follows:

(1) $$E_{k}=-\sum_{i,j\in L}p_{k}\left(x_{ij}\right)logp_{k}\left(x_{ij}\right)$$

One can see that the value of entropy may provide an indication of the degree to which students interact with the learning system. In scenarios where a student’s entropy is zero, this reflects a complete lack of engagement and the absence of any observed learning activity. Conversely, a student showing a high entropy value is suggestive of substantial engagement in numerous learning activities. Investigating the correlation between entropy-based metrics, regarded as student learning characteristics, and students’ academic performance has the potential to reveal insights into the impact of engagement levels on academic outcomes and the identification of patterns that could enable early intervention for students at risk of underperformance.

### 4.2. Coefficient of Variation of Entropy

It would be normal for a student’s entropy to vary daily, contingent upon the quantity of learning material items the student engages with on a given day and the amount of time the student devotes to the course. Therefore, it can be useful to examine the variability of a student’s entropy throughout the learning period.

However, the computation of entropy can be sensitive to the number of learning items employed by a student. Furthermore, each student may adhere to distinct personal learning styles. To compare in an equitable way how entropy varies from person to person, we utilise the concept of the *coefficient of variation of entropy*, denoted as CoV. The computation of CoV of a student is as follows:

(2) $$CoV_{i}^{k}=\frac{\sigma_{i}^{k}}{\mu_{i}^{k}}$$

where $CoV_{i}$ refers to the coefficient of variation of entropy for student *k* until day *i*, while $\sigma_{i}^{k}$ and $\mu_{i}^{k}$ are the standard deviation and mean of entropy of the student *k* until day *i*, respectively.

In order to identify the student communities, i.e., the communities with students showing successful learning patterns and vice versa, we utilised the method proposed in [18], which is based on random matrix theory and community detection. This approach has demonstrated success in handling the noise and trend problem in similar students’ learning log data. The following sections show our adoption of the method in the context of this research.

### 4.3. Random Matrix Theory

Given an m×n data matrix G extracted from an event log, the matrix G(n) can be normalized as follows [53]:

(3) $$G{\left(n\right)}_{j}=\frac{G_{j}-\bar{G_{j}}}{\sigma_{j}}$$

where $G{\left(n\right)}_{j}$ is the jth column of the matrix G(n); $G_{j}$ is the jth column of the matrix G. In the case where G is *a transition–student data matrix*, $G_{j}$ denotes the frequency of all occurred transitions of a student *j*. For instance, in Table 2, $G_{j}$ refers to column s1, s2, etc. $\bar{G_{j}}$ is the mean value of $G_{j}$ and $\sigma_{j}$ is the standard deviation of $G_{j}$. In other words, $G_{j}$ and $G{\left(n\right)}_{j}$ reflect the learning behaviour of the student *j*.

The correlation matrix C can be expressed in terms of the inner product of $G{\left(n\right)}_{i}$ and $G{\left(n\right)}_{j}$ as follows:

(4) $$C_{ij}=\langle G{\left(n\right)}_{i},G{\left(n\right)}_{j}\rangle$$

We note that $C_{ij}\in \left[-1;1\right]$. It may be noticed that the correlation $C_{ij}$ can reflect how similarly two students *i* and *j* interacted with course material items. If $C_{ij}>0$, the transitions of the two students *i* and *j* increased together and the students behaved similarly in the course. Conversely, if $C_{ij}<0$, the two students tend to behave differently on the learning system.

The eigendecomposition of C can be shown (e.g., see [53]) to be given by

(5) CV=ΛV

where Λ is a diagonal matrix *n* × *n* of eigenvalues $\lambda_{i}$ and V is a matrix whose columns refer to the corresponding eigenvectors ${\mathrm{vi}}_{i}$ of C.

Given a matrix A where A is a matrix m×n with randomly distributed elements with zero mean and unit variance, it has been shown [54] that the properties of C can be compared to the correlation matrix R of the random matrix A as

(6) $$R=\frac{1}{m}{\mathrm{AA}}^{T}$$

where $A^{T}$ is the transposed matrix of A. This briefly states random matrix theory (RMT). Based on RMT, the statistical properties of such a matrix R can be determined [55]. When the sample size m→∞ and the number of features n→∞, provided that Q-factor $=\frac{m}{n}\geq 1$ remains unchanged, the distribution of eigenvalues λ of the random matrix R can be determined by the Marchenko–Pastur probability density function as follows [56]:

(7) $$P_{R}\left(\lambda \right)=\frac{Q}{2\pi \sigma^{2}}\frac{\sqrt{\left(\lambda_{+}-\lambda \right)\left(\lambda -\lambda_{-}\right)}}{\lambda }$$

where $\lambda_{-}\leq \lambda \leq \lambda_{+}$, $\lambda_{-}$ and $\lambda_{+}$ are the lower and upper limits, the eigenvalues of R, respectively, given by

(8) $$\lambda_{\pm }=\sigma^{2}{\left(1\pm \sqrt{\frac{1}{Q}}\right)}^{2}$$

where σ=1 due to A having unit variance.

We note that $\lambda_{\pm }$ are the upper/lower limits of the theoretical eigenvalue distribution. Eigenvalues that fall outside of this range are assumed to deviate from the expected values of the RMT [57] and potentially contain information. Hence, by comparing this theoretical distribution with the empirical data, we can identify key eigenvalues containing specific information on the data. This characteristic of the RMT supports the need to clean the effect of noise and trend in the data [56,58].

### 4.4. Noise and Trend Effect Cleaning

It has been noticed [18,59] that, in practical usage of the online learning system, students tend to interact sporadically with course material items. This phenomenon appears to create noise in the data and may limit the chance of detecting the difference in learning behaviours among students. Therefore, it is necessary to “clean” the noise effect in the data [56].

In this research, we utilise the noise cleaning method known as *eigenvalue clipping* because of its ability to remove the noise while preserving the information part, i.e., maintaining the trace of the original correlation matrix, by simply utilising the results of the Marchenko–Pastur equation mentioned above [60] instead of choosing a parameter during the cleaning process, such as *linear shrinkage* and *rotationally invariant optimal shrinkage* [61]. The *eigenvalue clipping* provides robust out-of-sample performance [62] and has also been widely adopted [63].

Let $\lambda_{1},\ldots ,\lambda_{N}$ be the set of all eigenvalues of C and $\lambda_{1}>\cdots >\lambda_{N}$, and *i* be the position of the eigenvalue such that $\lambda_{i}>\lambda_{+}$ and $\lambda_{i+1}>\lambda_{+}$.

Then, we set

(9) $$\lambda_{j}=1/\left(N-i\right)\sum_{k=i+1}^{N}\lambda_{k},$$

where j=i+1,…,N. In other words, we keep all the upper bound eigenvalues, i.e., those with information, and replace all lower bound eigenvalues, i.e., those within bounds predicted by RMT, with the average value of them. Hence, this method can preserve the trace of the original correlation matrix. The new set of eigenvalues can be used to construct a denoised eigenvalue and spectrum-associated correlation matrix $C_{denoised}$ [56].

We observe a phenomenon that students’ learning behaviours can be affected by a trend factor, i.e., they were asked to follow the same instructions and learning pathway, causing highly positively correlated learning behaviours among the students. By removing such a trend component, the remaining components of the correlation could explain better the characteristics of the students’ learning behaviours. Therefore, we adopt, from financial references such as [64], the concept of a “Market Component”. This is the largest eigenvalue of a financial correlation matrix representing a cross-market effect affecting all stocks. Similarly, the trend effect in a classroom can be reflected by the largest eigenvalue of the correlation matrix C. The effect of the first eigenvalue and eigenvector can be removed from the denoised correlation matrix as follows [56], forming a cleaned correlation matrix:

(10) $$C_{cleaned}=C_{denoised}-W_{1}\lambda_{1}W_{1}^{T}$$

where $W_{1}$ and $\lambda_{1}$ are the first eigenvector and eigenvalue of *C*.

### 4.5. Distance of Learning Behaviours between Students

While correlation matrices have been commonly used to reflect similarities and differences in students’ learning behaviours, they possess limitations as appropriate metrics due to their failure to satisfy non-negativity and triangle inequality conditions [56]. The lack of adherence to these conditions renders the values of the entries less suitable for capturing meaningful relationships accurately. For instance, the difference between correlation tuples (0.8, 1.0) is considered equivalent to that of (0.1, 0.3) despite the former implying a higher divergence concerning co-dependence. As a result, alternative metrics are sought to provide more meaningful representations of students’ learning behaviours.

Fortunately, the conversion of correlations into a distance matrix D can be achieved as follows [18]:

(11) $$D_{ij}=\sqrt{0.5*(1-C_{ij})}$$

with $D_{ij}\in \left[0,1\right]$, where $D_{ij}$ is a distance value of learning behaviours between two students *i* and *j*. A value close to 1 in the distance matrix signifies that the two students interact with the course material items in significantly distinct ways. Conversely, a value closer to 0 indicates that the two students exhibit similar learning behaviours, demonstrating a higher degree of similarity in their interactions with the course materials.

### 4.6. Constructing the Graph of Students’ Learning Behaviours

The main purpose of community detection is to verify whether students with similar learning behaviours perform differently in lab exams. In order to achieve this, we adopt a network-based approach. In the first step, based on the distance matrix extracted from the *transition–student data matrix*, we construct the minimum spanning tree (MST) [65], which connects all students without having any loops.

If we consider the distance matrix D as the adjacency matrix of a graph, the MST is constructed in such a way that the sum of all edges in the graph is minimal for all possible spanning trees from the graph based on the adjacency matrix D. It can be seen that the MST of a set of *n* students can be represented by a graph with n−1 edges. Each student can be connected to one or more other students who have the most similar behaviours to that student, which is based on the premise that the distance matrix measures the similarities in learning behaviours between students. In this way, the clustering purpose is preserved.

### 4.7. Community Detection on the MST Graph

Using the MST constructed from the distance matrix, it is possible to advance to the next step, i.e., community detection (CD), which is supported by several methods [66,67]. In this paper, we utilise the popular detection algorithm from Girvan and Newman [67], which is applied in various domains, such as biology [67] and finance [68]. In fact, other commonly used CD methods exist (e.g., Louvain algorithm), but their application has been found here not to affect the clustering results in a large way [18].

The Girvan–Newman algorithm aims to divide the whole network into smaller communities or groups by progressively removing edges with the highest *betweenness* until no edges are remaining. *Betweenness* is the number of the shortest paths between pairs of nodes that run through it from the original network [67]. The nodes of students in a smaller group connect more to each other than the ones outside the group. In other words, students in the same group performed a similar behaviour in using learning material items.

## 5. Results

### 5.1. Selecting Community Structure

Details of the results for the community detection of each dataset can be viewed in Table 4. In each dataset, eight groups have been detected, with the number of students in each group and their average grades for the final lab exam in week 12 (end of Module-2018 and Module-2019) and in week 10 (end of Module-2020). All groups are ordered from the highest to the lowest average grades in the tables. Given that students in a detected group have similar learning behaviours, we conclude that there is a relationship between students’ learning behaviours and learning outcomes. Generally, students having similar learning behaviours are grouped in a community. We notice from the communities detected that some groups mostly include higher-performing students (based on their grades in the final lab exam), while other groups mostly contain lower-performing students.

Once the communities are detected, we can focus more on representative communities to figure out the study pattern of students in using of learning material items. In the sections below, with analyses using entropy-based metrics, we will compare the learning patterns between higher-performing communities—containing students from the top four groups in Table 4—and lower-performing communities—containing students from the last four groups in Table 4.

### 5.2. Representing Learning Progress Using Entropy

**Figure 1:**
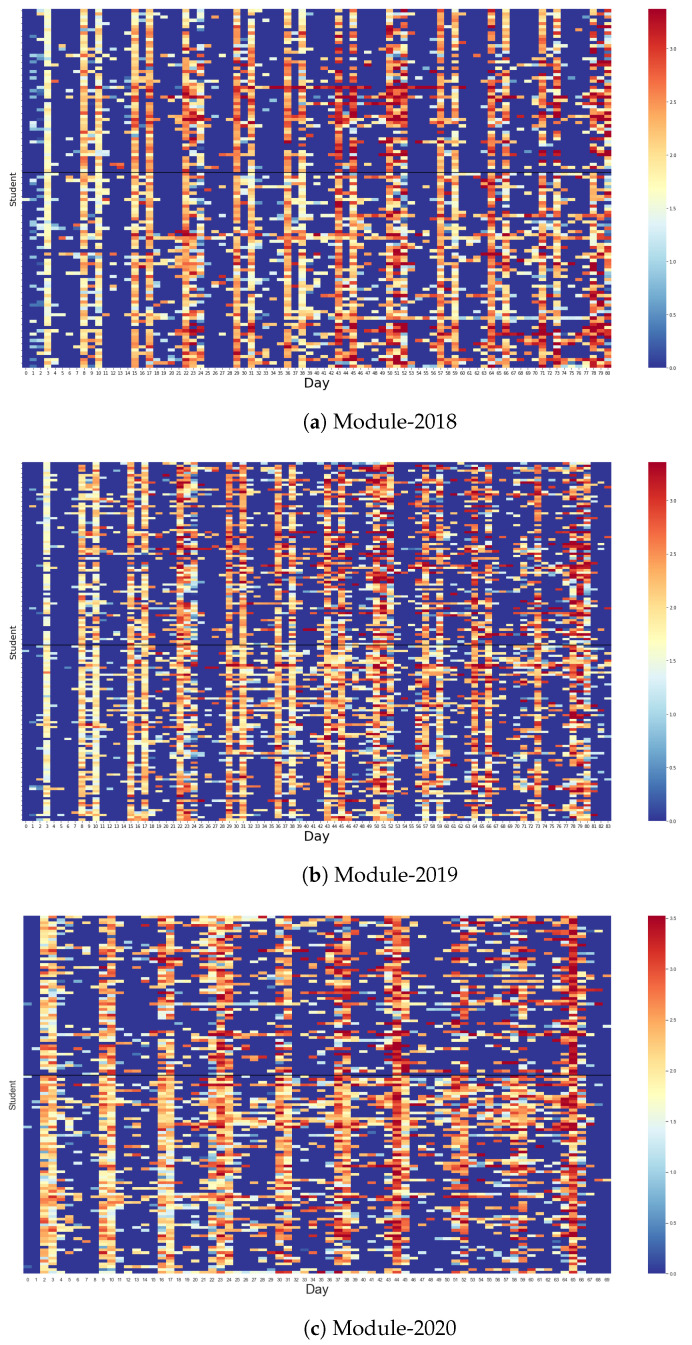
The heat maps show the entropy values for each student on each day in their programming courses. Warmer colours (more red) suggest higher entropy values and more active learning activities, while cooler colours (more blue) indicate lower entropy values and less active learning behaviours. In each figure, within every seven days, typically two days emerge as significantly more active than the rest, as evidenced by the majority of students exhibiting higher entropy values. This pattern aligns with the instructional schedule, wherein students typically dedicate one day to lecture sessions and another day to practical exercises in the lab. On non-scheduled learning days, a subset of students displays no activity, as evidenced by zero entropy values and the resultant plain blue hue on the heat maps.

Figure 1 presents three heat maps that display the daily entropy values for each student in all three programming courses. These entropy values, represented on a colour spectrum, indicate the degree of unpredictability in students’ learning behaviours. Higher entropy values, denoted by warmer colours, suggest a greater diversity in learning activities, while lower values, depicted in cooler colours, indicate less varied learning behaviours. The *x*-axis of the heat map denotes each day of the semester, and the *y*-axis corresponds to individual students.

A weekly pattern is discernible across all courses from the heat maps. Within every seven-day period, two days emerge as significantly more active than the rest, as evidenced by the majority of students exhibiting higher entropy values. This pattern aligns with the instructional schedule, wherein students typically dedicate one day to lecture sessions and another day to practical exercises in the lab.

However, this pattern deviates in the first week of both Module-2018 and Module-2019, as depicted in Figure 1a,b. In both instances, only one active day is observed in the first week. This suggests that the initial lab session, typically scheduled for Tuesdays and Thursdays, was omitted, and the course commenced with theoretical lessons on Thursday. In contrast, Module-2020, which was conducted during the COVID-19 pandemic over a reduced period of 10 weeks, exhibits a different pattern. In this course, lecture and lab sessions were scheduled on Wednesday and Thursday, respectively, and all scheduled studying days were utilised during the course, as visible in Figure 1c.

On non-scheduled learning days, a subset of students displays no activity, as evidenced by zero entropy values and the resultant plain blue hue on the heat maps. However, a number of students still demonstrate engagement with learning activities on these non-scheduled days, albeit at a reduced intensity.

Overall, the entropy values visualised in these heat maps offer an effective representation of the progression of students’ learning. In particular, they appear to show that, as has been pointed out by Figure 1, there is evidence of an increased pattern of interaction with the learning system in Module-2020 (during lockdown) over the previous years, albeit there has been a trend underway from 2018 to 2019. The heatmaps encapsulate the variability in students’ engagement with diverse learning activities and their adaptation to the instructional schedule. These entropy measures provide an insightful lens to understand and monitor students’ learning behaviours and engagement patterns in programming courses.

Nevertheless, discerning the differences in learning behaviours among various student communities using the aforementioned heat maps can prove challenging due to the high variability in entropy values. To address this limitation, we conducted a comparative analysis of the proportion of active students, i.e., students with non-zero entropy values, within each community on each day during the semester. Additionally, we employ the statistical measure of the coefficient of variation in entropy values to provide further insights into the dispersion of students’ learning behaviours. The outcomes of these complementary analyses will be elucidated in the subsequent section.

### 5.3. Similarities and Differences in Learning Behaviours Represented in Entropy-Based Metrics

**Figure 2:**
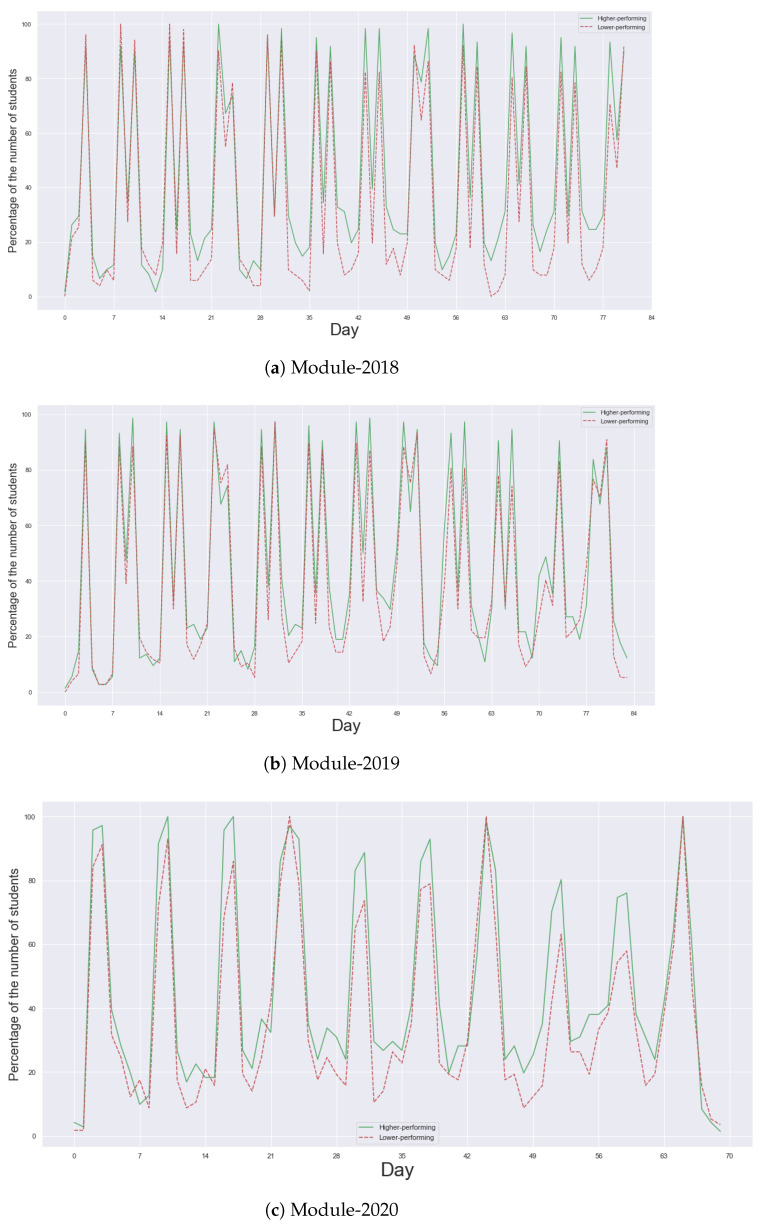
The percentage of students having positive learning behavioural entropy values in three years of the course. Within each week, a substantial majority of students—typically exceeding 80%—in both higher- and lower-performing communities were observed to be actively engaged on lecture and practice days. The higher-performing communities (green lines) consistently display a higher percentage of active students, particularly on non-scheduled studying days, in comparison with that of the lower-performing communities (red lines).

In alignment with the patterns observed in the heatmaps in Figure 1, Figure 2 shows a clear weekly rhythm in students’ learning activities. Within each week, a substantial majority of students—typically exceeding 80%—in both higher- and lower-performing communities were observed to be actively engaged on lecture and practice days. This pattern underscores the influence of the scheduled teaching plan on students’ learning behaviours.

Figure 2 also provides a representation of the percentage of students exhibiting positive entropy values each day in their programming courses, distinguishing between higher-performing and lower-performing communities. We assume that positive entropy values are indicative of engagement in diverse learning activities, thus providing a measure of students’ active participation in their coursework. An intriguing distinction emerges when comparing the higher-performing communities to their lower-performing counterparts. The former consistently displays a higher percentage of active students, particularly on non-scheduled studying days. This suggests that higher-performing students may be more likely to engage in self-directed learning outside of the scheduled teaching activities, potentially contributing to their superior academic performance.

On non-studying days, however, the proportion of students engaging with learning materials significantly drops, averaging around 20% across the three programming courses. This demonstrates a clear dichotomy in the learning behaviours of students between scheduled and non-scheduled studying days.

These findings, captured through the application of entropy as a measure of learning behaviours, provide valuable insights into the patterns and strategies that distinguish higher- and lower-performing students in programming courses. Such understanding can guide pedagogical strategies to better support and enhance students’ learning experiences.

**Figure 3:**
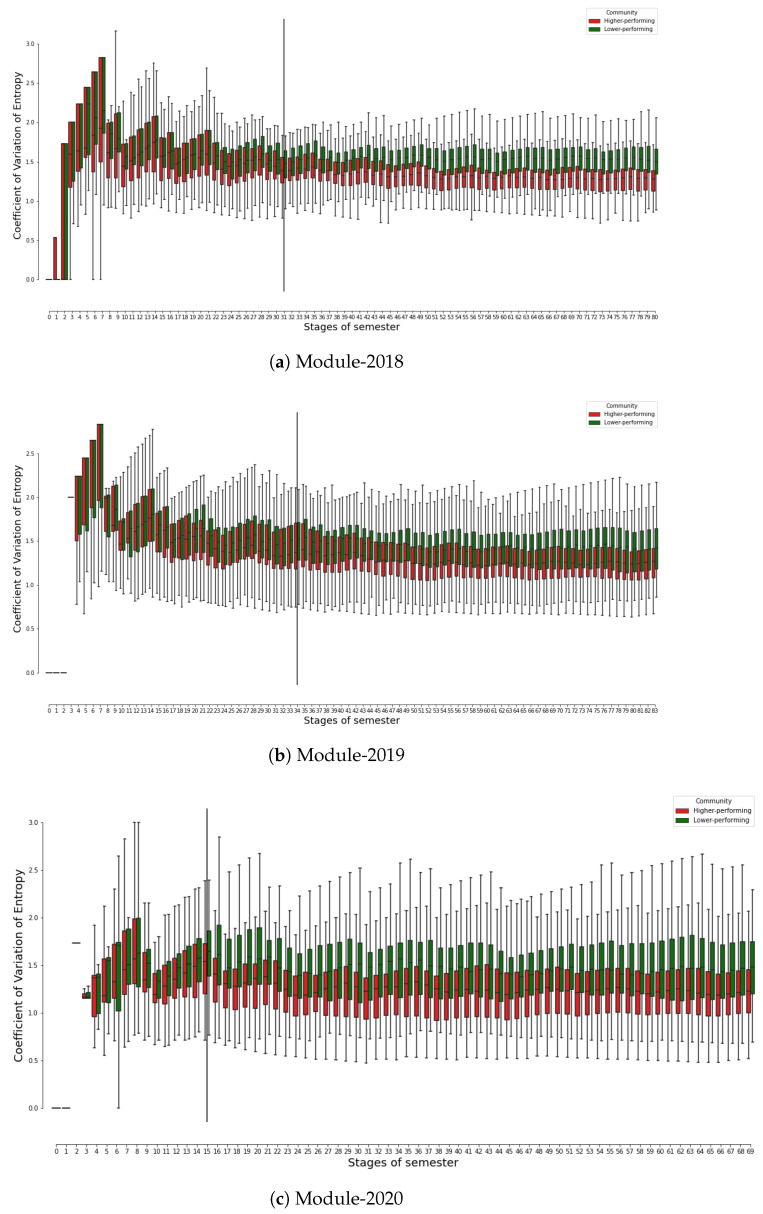
The distribution of the coefficient of variation of entropy by higher- and lower-performing student communities across three courses. Learning dynamics present higher coefficients of variation in early stages, which decrease as courses progress. Lower-performing students (green bars) show higher entropy variation than higher-performing ones (red bars). Higher-performing communities stabilise earlier (red bars) than lower-performing communities (green bars). The vertical solid lines mark the “split-up day”—meaning that, in the following days, the statically significant differences between the two communities have been found.

Figure 3 comprises three boxplot graphs, each illustrating the distribution of the coefficient of variation of entropy values for higher- and lower-performing student communities in three courses. Please note that the coefficient of variation of entropy for a student on a given day, *n*, is calculated based on the entropy values of the student from the commencement of the course (Day 0) until the end of Day *n*, as detailed in Section 3.2.

From a broad perspective, all three years of the course show a comparable pattern. Initially, higher coefficients of variation are typically observed in the early stages of the courses, indicative of an evolving learning dynamic. As the courses progress into the middle and later phases, the coefficient values tend to decrease and stabilise, suggesting that students’ learning behaviours become more consistent over time.

Figure 3 also shows that the better-performing student communities settle down to a stable level, i.e., reaching the lower values of the coefficient of entropy variation earlier than the lower-performing communities. This pattern potentially signals a transitional phase in which students are acclimating to the course and familiarising themselves with the learning materials. Such a period of adjustment is to be expected at the onset of a course as students navigate the learning environment and resources.

Interestingly, Figure 3 also indicates the difference in the coefficient of variation of entropy between higher- and lower-performing communities. It is of particular interest to note that students belonging to the lower-performing communities (denoted by green boxes) appear to have a higher coefficient of variation of entropy in comparison to their counterparts in the higher-performing communities (represented by red boxes). This observation is statistically substantiated by the Mann–Whitney U tests, which assess the difference in the coefficient of variation of entropy values between the two communities for each day of the course.

In particular, during Module-2018, no statistically significant disparity in the coefficient of variation of entropy is observed between the higher- and lower-performing communities up until day 31. Following this so-called “split-up day”, the divergence between the two communities begins to escalate over time, evidenced by a *p*-value of less than 0.05 for all subsequent days until the culmination of the course.

A similar trend is also observed in both Module-2019 and Module-2020, with the “split-up day” manifesting on Day 34 and Day 15, respectively, as indicated by the solid vertical lines on the corresponding graphs.

It is also essential to highlight that the “split-up day” for Module-2020 occurs considerably earlier than in the other two courses. This can be attributed to the exceptional circumstances of Module-2020, which was conducted during the COVID-19 pandemic with a reduced course duration (10 weeks as opposed to the typical 12 weeks). The abbreviated timeframe may have necessitated an earlier divergence in learning behaviours between the two communities despite the comparable level of learning and assessments across the three academic years. Anecdotally, the length of the semester—at 10 weeks—did not appeal to either the bulk of students or lecturers on the course as both found it insufficient to master the concepts.

The implication of these findings may be that higher-performing students demonstrate greater consistency in their study habits, whereas lower-performing students might encounter more challenges and inconsistency in managing the learning requirements, thereby resulting in a higher degree of variability in their learning behaviours. Such insights into students’ learning dynamics offer valuable direction for tailored pedagogical interventions.

**Figure 4:**
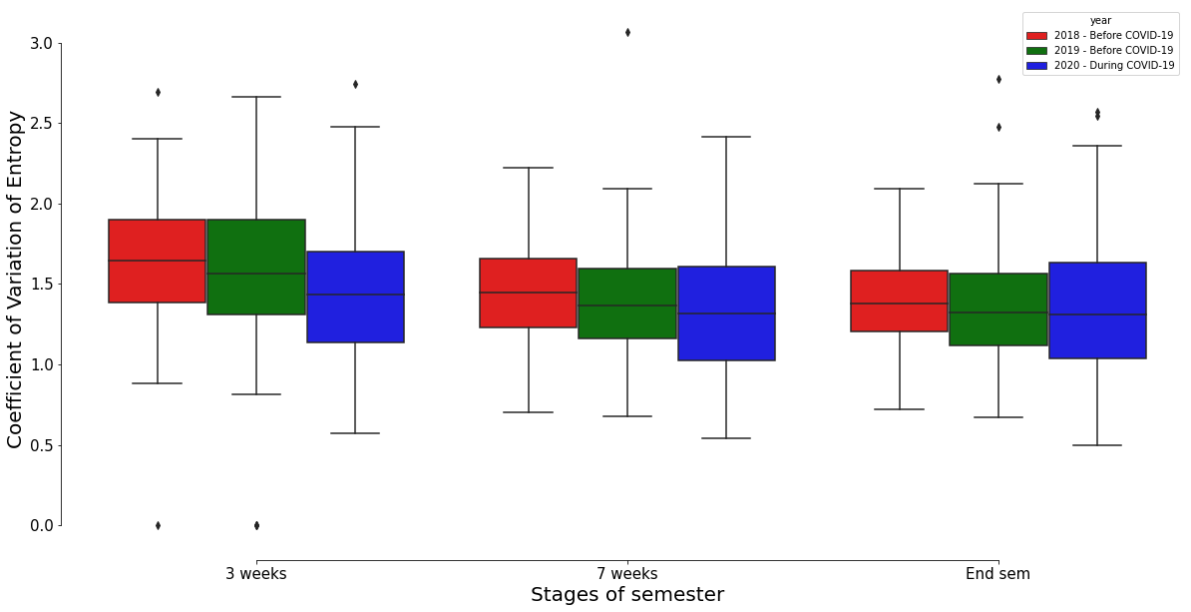
The distribution of the coefficient of variation of entropy for the courses before and during the COVID-19-pandemic. Note that the End semester phase refers to the end point of the course, i.e., after week 12 with Module-2018 and Module-2019, and after week 10 with Module-2020.

In Figure 4, a boxplot is used to juxtapose the distributions of the coefficient of variation of entropy across the three programming courses at varying stages of the semester, i.e., after 3 weeks, 7 weeks, and at the end of the courses. This allows for a comprehensive comparison of student learning behaviours, as encapsulated by the entropy metric, across different time points in the courses.

Upon conducting the Mann–Whitney U tests, a non-parametric statistical hypothesis test, we found no statistically significant disparities in the distributions of the coefficient of variation of entropy across the three academic years of the course at any stage of the semester (*p*-value > 0.05). This suggests that the differences in entropy values observed between the courses do not reach statistical significance, implying that student learning behaviours, as reflected by their entropy values, were consistent across the three courses regardless of the stage of the semester.

This finding carries particular relevance for Module-2020, which was conducted during the COVID-19 pandemic. Despite the challenging circumstances and the altered educational landscape brought about by the pandemic, it appears that Module-2020 was adeptly organised such that students could effectively engage with the learning materials. This is evidenced by the consistent entropy values, suggesting that the students’ learning behaviours in Module-2020 remained on par with those observed in the other courses, irrespective of the pandemic conditions.

## 6. Discussion

### 6.1. Research Question 1 Revisit: Representing the Students Learning Behaviours Using the Concept of Entropy

The analysis of entropy in the context of students’ learning behaviours in programming courses has proven to be an effective method for understanding these behaviours, as highlighted in Section 5. The concept of entropy, originally derived from thermodynamics and information theory, provides a novel perspective on student engagement and learning progress in this study. Our findings align with the premise proposed in [17,52], where the authors asserted that entropy could be a measure of uncertainty or disorder within a system, and, in our case, the system is the learning process of students programming on a module.

Our research demonstrates that the entropy measure can serve as a reliable and straightforward indicator of student learning behaviours, allowing educators to efficiently monitor the studying progress in a class of students. This is particularly useful in EDM/LA, where multiple features often obscure the broader picture. Through entropy measurement, educators can assess the level of student engagement by tracking the percentage of students with positive entropy values. This is in line with [13,69,70], who affirmed the importance of monitoring student engagement in virtual learning environments, suggesting the use of data mining techniques to track student behaviours. Our study extends this proposition by introducing entropy as a simplified, yet insightful, metric.

A particularly intriguing insight from our research is the potential for the coefficient of variation of entropy to act as a marker of student learning progress. A persistently high coefficient may signify difficulties that students are encountering with learning materials. This has the potential to offer a valuable tool for educators, allowing them to identify and intervene when students are experiencing challenges. This finding complements previous research [71] in which the authors showed the importance of early detection of students’ learning struggles in improving learning outcomes.

### 6.2. Research Question 2 Revisit: Learning Behaviour Entropy between Higher- and Lower-Performance Student Communities

The analysis of entropy as a distinguishing characteristic between higher-performing and lower-performing student communities presents an intriguing finding in our study. The use of entropy-based metrics to represent student performance aligns with the existing body of literature that emphasises the significance of understanding student learning behaviours in enhancing educational outcomes [72].

Our results demonstrate that lower-performing students tend to exhibit either zero values, indicative of an idle status, or higher values of the coefficient of variation of entropy, suggestive of a higher volatility of entropy in each student on average, and potential struggles in their learning process. These findings are in line with [73] in which the authors indicated the importance of identifying at-risk students for early interventions. In contrast, higher-performing students in our study exhibited lower values of the coefficient of variation of entropy, showing more stable learning patterns. This is consistent with previous works [74,75] that have demonstrated a positive correlation between consistent engagement in learning activities and better academic performance.

The implications of our findings extend to the pedagogical practices, suggesting that educators can leverage these entropy-based metrics to target student interventions more effectively. For example, by monitoring the coefficient of entropy variation, educators can cluster and identify students with extremely high or low and unstable values, indicating a need for intervention. Even from Figure 3, one may notice that those whose coefficient of entropy variation does not increase monotonically in the first seven days can be defined as early lagging students. By promptly identifying and addressing these students’ needs, educators can potentially enhance the overall effectiveness of teaching and learning processes.

Our research highlights the potential utility of entropy in EDM/LA. The insights gained about higher-performing and lower-performing student communities through entropy-based metrics can inform more effective pedagogical strategies. Future research may aim to further validate these findings across different educational contexts and explore the integration of entropy with other data mining techniques for a more comprehensive analysis of students’ learning behaviours.

### 6.3. Research Question 3 Revisit: Using Entropy-Based Metrics as Dynamic Indexes to Monitor the Students’ Level of Engagement during the Studying Time

Based on the analysis result, we can see that entropy-based metrics have obvious potential to be used as dynamic, time-dependent indicators to monitor and address students’ learning engagement and performance challenges in real time. By continuously tracking and analysing the entropy values (e.g., on a daily basis, as can be seen in Figure 3), educators can gain insights into the evolving patterns of individual student engagement and adjust their instructional strategies in a targeted way for individualised and personalised attention to meet their learning needs. Such analysis can assist in identifying potential issues early, and enable educators to intervene timely to mitigate any adverse effects on student learning.

Entropy-based indicators such as coefficient of variation of entropy can serve as a simple and effective metric for educators to swiftly evaluate the state of learning in a course. Educators can compare the metric across different academic years to monitor the continuity or changes in learning behaviours. This may help to identify students who consistently exhibit low engagement or high volatility in their learning behaviours. Based on this information, educators can create personalised learning paths tailored to individual needs. For students with lower engagement, more interactive and engaging learning materials could be provided, while, for those with higher volatility, additional support and reinforcement of key concepts might be necessary.

Entropy-based metrics can also be integrated into learning management systems to provide real-time feedback to educators about students’ engagement levels and behaviours. For example, educators can identify students at risk of disengagement or inconsistent learning behaviours early on. This information can pave the way for timely interventions and support mechanisms for “at-risk” students, such as sending reminders, offering additional resources, or scheduling one-on-one sessions to address specific challenges. Furthermore, learning content can also be adapted based on its effectiveness in engaging students. Learning materials that consistently lead to higher engagement and lower volatility can be prioritised, while less effective materials can be re-evaluated or replaced.

In terms of teacher training and development, entropy-based metrics can also be used to assess the effectiveness of educators in engaging their students. Educators or lecturers with consistently high entropy values across their classes might benefit from additional training and support in instructional strategies to improve engagement and consistency. In addition, tracking entropy-based metrics over a substantial period can help to assess the overall effectiveness of the instructional approach. For example, if the volatility of entropy values remains stable over time, it might indicate that the instructional methods are ensuring sustained engagement and consistent learning behaviours. Otherwise, educators may need to re-assess their teaching strategies.

Overall, we envision that our results provide material for further work to understand how motivational interventions can be optimised to improve educational outcomes among those individuals who need them most, as has been recommended by, e.g., [76]. In addition, in order for the research to be rigorous (as found by [77], for example), it is necessary to include as many contributory factors as possible (e.g., socio-economic status, on-campus vs off-campus study mode, part-time vs full-time student cohorts, etc.). As our dataset is anonymised, many of these individual student characteristics are lost. It is for this reason that we incorporate data from 2020, when all students were studying off campus due to COVID-19, to investigate whether trends in learning behaviours observed in previous years were continued. The apparent trend into that year of continuous—if not increased—student engagement with the course through the learning management system was an interesting finding, which requires further investigation to substantiate it.

In summary, entropy presents as a valuable tool for educators, offering a relatively simple but effective metric for monitoring students’ learning progress. This study contributes to the emerging field of EDM/LA by advocating for the application of entropy in assessing student engagement and progress. However, there are some challenges and limitations in applying entropy-based metrics in education, such as:

- The choice of appropriate entropy measures and data sources for different learning contexts and objectives.
- The interpretation and communication of entropy-based metrics to teachers and students in a meaningful and actionable way.
- The ethical and privacy issues related to collecting and analysing behavioural data from students.

These challenges require further research and development to make entropy-based metrics more accessible and useful for education. Future research may expand upon this preliminary study, potentially utilising entropy in conjunction with other data mining techniques to offer a more comprehensive analysis of students’ learning behaviours in programming courses and other educational settings.

## 7. Conclusions

This research has demonstrated the applicability of the concept of entropy for the comprehensive analysis of students’ learning behaviours. Utilising behavioural data collected from 391 Software Engineering students across three academic years, including periods before and during the COVID-19 pandemic, we were able to extract insightful metrics related to learning behaviour based on the concept of entropy.

Particularly, we proposed an innovative approach to extract entropy-based metrics from students’ learning log data. These metrics allowed for a nuanced investigation into the variations in learning behaviours across different student communities and academic years. The communities were established based on academic performance, as determined by a method proposed in the literature. This method was particularly useful in mitigating the impact of noise and trends in the log data, ensuring a more reliable clustering of students.

Our findings revealed distinct patterns in students’ learning behaviours. Generally, students were predominantly active on days scheduled for studying and practising. However, on non-scheduled days, the active student population was significantly smaller. This pattern was more pronounced amongst higher-performing students, who demonstrated a greater propensity for engagement, particularly on non-scheduled study days.

The coefficient of variation of entropy emerged as a particularly useful metric in differentiating between the higher- and lower-performing communities. We observed that students in lower-performing communities exhibited a higher coefficient of variation of entropy, signifying greater variability in their learning behaviours.

Overall, our research underscores the potential of entropy as a valuable metric in the context of educational research. It serves as a simple but effective tool for educators to monitor students’ learning progression and to identify potential issues that may require timely intervention. In line with this starting point, our future work aims to explore the applicability of diverse entropy types within the context of educational data. Additionally, we plan to investigate the potential for entropy-based metrics to monitor chronological changes in student learning behaviours over his/her academic journey. This study paves the way for future research to explore more nuanced applications of entropy-based metrics in analysing and enhancing students’ learning behaviours in various educational settings.

## Figures and Tables

**Table 1:** Datasets information.

Dataset	Number of Students	Number of Events	Average Events per Student
Module-2018	112	1,054,394	9414
Module-2019	151	1,484,297	9829
Module-2020	128	1,589,216	12,415

**Table 2:** Example of transition–student data matrix.

Transition	s1	s2	s3	s4	⋯
Lecture1-Lecture1	4	5	10	23	⋯
Lecture1-Labsheet1	0	14	9	12	⋯
Labsheet1-Practice1	12	6	0	21	⋯
⋯	⋯	⋯	⋯	⋯	⋯

**Table 3:** Details of the datasets for community detection analysis.

Dataset	Number of Students (Columns)	Number of Transitions (Rows)	Number of Higher-Performing Students	Number of Lower-Performing Students
Module-2018	112	825	54	58
Module-2019	151	878	87	64
Module-2020	128	602	69	59

**Table 4:** Community detection summary for Module. Groups are ordered in descending order based on the average grades in the terminal assessment of their members.

	Module-2018		Module-2019		Module-2020	
	**Number of Students**	**Average Grade**	**Number of Students**	**Average Grade**	**Number of Students**	**Average Grade**
Group 1	18	**0.79**	12	**0.89**	19	**0.71**
Group 2	11	0.52	16	0.64	18	0.59
Group 3	15	0.5	21	0.61	19	0.56
Group 4	17	0.42	25	0.57	15	0.43
Group 5	11	0.25	17	0.32	19	0.42
Group 6	13	0.21	14	0.32	11	0.38
Group 7	13	0.17	20	0.31	15	0.36
Group 8	14	**0.05**	26	**0.25**	12	**0.08**

## Data Availability

Data is unavailable due to privacy and ethical restrictions.
